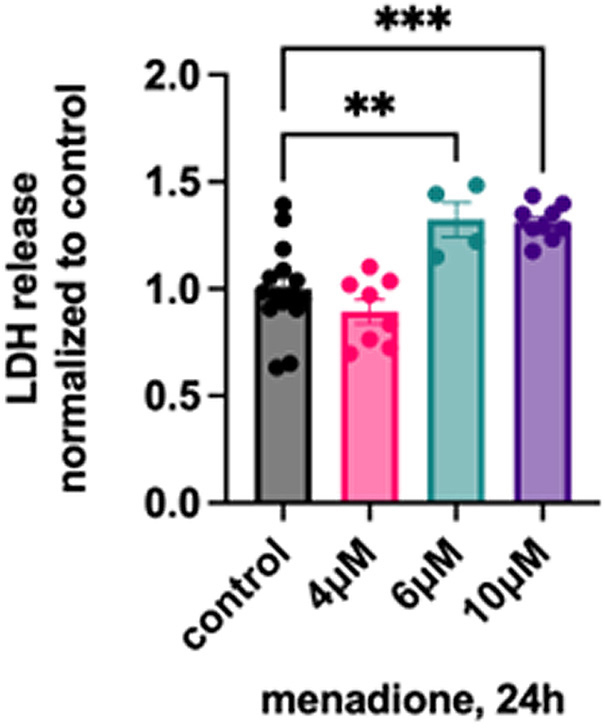# Potent antioxidant and mitochondrial-protective effects of ATH434, a moderate affinity iron chaperone

**DOI:** 10.1016/j.jbc.2025.110595

**Published:** 2025-08-13

**Authors:** Danielle K. Bailey, Rhudwan Nihlawi, Margaret J. Bradbury, Silas Bond, Daniel J. Kosman

**Affiliations:** 1 Department of Biochemistry, State University of New York at Buffalo, Jacobs School of Medicine and Biomedical Sciences, Buffalo, New York, USA; 2 Alterity Therapeutics, Newark, California, USA; 3 Alterity Therapeutics Limited, Melbourne, Victoria, Australia

**Keywords:** ATH434, antioxidant, mitochondrial protection, oxidative stress, iron chaperone, neurodegeneration, brain iron accumulation

## Abstract

A plethora of neurologic disease presents with brain iron accumulation. Among these disorders are Parkinson’s disease (PD) and Multiple System Atrophy (MSA). Development of interventions logically has focused on the iron-dependent oxidant stress characteristic of these pathologies. This focus is represented using iron chelators on the one hand and antioxidant therapy on the other. Here we provide evidence that ATH434, a small-molecule drug candidate with similar and moderate affinity for both ferrous and ferric iron (*K*_d_ ∼10^−6^), exhibits both electron (ET) and hydrogen atom transfer (HAT) antioxidant activity. Previous studies have shown that ATH434 promotes cellular iron efflux, reduces excess brain iron and aggregated α-synuclein, improves neuronal survival, and restores motor performance in murine PD and MSA models. ATH434 has been granted Orphan drug and Fast Track designation by the FDA. Here we have established that ATH434 is a robust antioxidant. We have demonstrated that it protects mitochondrial function and suppresses lipid hydroperoxidation in a menadione-induced model of oxidative stress in a glutamatergic neuronal cell line, HT22. Comparison of the electron transfer and hydrogen atom transfer activities of ATH434 with structurally related congeners provided insight into this compound’s structural features that support its varied antioxidant activity. These behaviors were consistent with the fact that cyclic voltammetry demonstrated ATH434 exhibits a chemically reversible electrode potential of 328.5 mV, unique to all antioxidants and iron chelators examined in this report. Our results indicate that ATH434 has the capacity to manage excess tissue iron and the oxidant stress induced by that iron.

The association of brain iron overload and oxidative stress with neurodegenerative disease has long been an important consideration for therapeutic intervention in these disorders. Iron has been associated with amyloid-beta plaques and tau tangles in Alzheimer’s Disease (AD) and in the alpha-synuclein aggregates in Parkinson’s Disease (PD) and Multiple System Atrophy (MSA) (1, 2, 3, 4, 5, 6, 7). Not only is this iron found associated with these proteins but it also appears to promote their aggregation, a characteristic of disease progression (7, 8, 9, 10, 11, 12). Brain iron accumulation is also linked to Friedreich’s Ataxia (FA) (13, 14) and Huntington’s Disease (HD) (15, 16) both of which have genetically linked downstream effects on iron homeostasis. While such links are not necessarily causative of disease, these correlations suggest the abnormal management and localization of iron in the brain, as well as the resultant oxidative stress associated with this iron, are hallmarks of such neurodegenerative disorders and represent an important target for pharmacologic intervention.

Iron chelation therapy for neurodegenerative disorders characterized by excess brain iron has had some success in suppressing pathological endpoints but commonly produces a worsening of symptoms that preclude their continued use. Deferiprone (DFP, Ferriprox), approved for systemic iron overload disorders like thalassemia and transfusion-induced anemia, has more recently been evaluated for efficacy in neurodegenerative disorders with brain iron accumulation. DFP has a *K*_d_ for Fe^3+^ of 10^−21^ M (17). In contrast, recent metal affinity competition studies quantified a ferrous iron *K*_d_ = 4.25 μM in anaerobic conditions at pH 7.5 (18). Thus, DFP favors Fe^3+^ over Fe^2+^ by a factor of 10^15^. The extreme difference in the stability of the ferric *versus* ferrous iron DFP complexes is responsible for the strong DFP-dependent potentiation of ferrous iron autooxidation and the generation of one-electron reactive oxygen species (ROS), including the superoxide anion radical (19).

While the ferric iron affinity of DFP is beneficial in the context of removal of excess iron from the circulation, it can be harmful intracellularly, where DFP can target iron prosthetic groups in essential Fe-containing proteins. For example, DFP inhibits Class 2 histone lysine demethylases (20). These demethylases are mono-nuclear iron dioxygenases similar in mechanism to prolyl hydroxylase domain enzymes (21). In a recent Phase 2 study, while DFP reduced brain iron as quantified by fMRI, the treatment accelerated cognitive decline (22). Similarly, a clinical trial of DFP in patients with PD who had never received levodopa (23, 24) reported reductions in brain iron and plasma ferritin - biomarkers of successful chelation of excess iron—but also noted a worsening of symptoms in a significant number of patients (23–25%) and severe drug-related neutropenia and agranulocytosis (24). These off-target effects appeared to be downstream of chelation of iron from tyrosine hydroxylase (TH), another mononuclear iron enzyme (25), thus reducing dopamine production further from the already low levels in patients with PD. DFP also targets the FeS cluster in aconitase (26). Deferasirox (DFX, Exjade), another iron chelator approved for chronic iron overload (27, 28, 29), has a similarly high ferric iron affinity and subsequent activation of iron autooxidation (18). In mouse models of AD, DFX decreases hyperphosphorylated tau (30) and reduces aging-related iron and amyloid-β accumulation (31), albeit without restoration of memory function (30).

In as much as ferrous iron is a pro-oxidant as reflected, for example, by the generation of the superoxide radical in its reaction with dioxygen, or in support of the Haber-Weiss/Fenton chemistry that generates the hydroxyl radical (32), antioxidant-based pharmacology represents an alternative or concurrent therapeutic intervention. Common nutraceutical antioxidants, such as ascorbic acid and cysteine/N-acetylcysteine, have been examined in treatment of degenerative neurologic disease (33, 34, 35). Idebenone (Catena), a CoQ_10_ analog, has been reported to improve FA symptom assessment scores and neurological symptoms but ultimately did not show improvement in cardiac function (36, 37). These mixed results are similar to trials of combined CoQ10 (ubiquinone) and Vitamin E (38, 39). Of note, co-treatment of patients having FA with idebenone and DFP led to a worsening of posture and gait scores (40). On the other hand, this combination did result in some improvement in cardiac function and other neurological parameters. Thus, there is evidence that combined antioxidant and iron-targeting therapies may be a promising therapeutic strategy. This approach recently has been highlighted in the demonstration that a resveratrol-DFP conjugate attenuates iron accumulation and oxidative stress in iron-challenged Huh7 cells (41).

ATH434 [(5,7-dichloro-2-((ethylamino)methyl)-8-hydroxy-3-methylquinazolin-4(3H)-one], a moderate affinity ferrous and ferric iron chelator (18), has been investigated as a potential therapeutic for PD, MSA, and other iron-related neurodegenerative disorders. ATH434 normalized disease-related increases in substantia nigra iron, reduced dopaminergic neuron loss in the substantia nigra, and supported a decrease in alpha-synuclein aggregation in oligodendrocytes in an MSA mouse model (42, 43). Similar effects were noted in a mouse model of PD in addition to reduced indices of oxidative stress (44). These activities have been attributed to the much lower affinity of ATH434 for Fe^3+^ in comparison to DFP or DFX. Thus, ATH434 exhibited a modest activation of ferrous iron’s prooxidant activity relative to DFP or DFX, and in the cellular milieu functioned as a ferrous iron chaperone in contrast to being a ferric iron chelator (18). The moderate affinity ATH434 has for Fe^2+^ (and for Fe^3+^) supports the redistribution of excess iron, supplementing the function of the cytoplasmic and nuclear PCBP1/2 iron chaperones (45). This property also limits competition by ATH434 for transferrin-bound iron (TBI) in the circulation (46). Recently, this compound successfully completed a Phase 2 clinical trial for MSA with primary goals of establishing biomarker, symptomatic efficacy, and safety profiles in patients (ClinicalTrials.gov).

In this report, we have investigated the differential chemical properties of ATH434 in comparison to DFP and DFX with a specific focus on the activity of these small-molecule therapeutics as antioxidants. Using an HT22 neuronal cell line model of oxidative stress induced by the radical generator menadione, we investigated the effects of ATH434 and comparator compounds on mitochondrial health by assessing mitochondrial membrane potential (MMP) using TMRM (tetramethylrhodamine methyl ester) staining. We also assessed their intracellular radical scavenging and suppression of downstream lipid peroxidation. To further investigate the mechanistic basis of these *in cellulo* impacts, we fully characterized the antioxidant capacity of our target compounds using in solution assays for their reactivity in both electron transfer (ET) and hydrogen atom transfer (HAT) reactions. The ability of ATH434 and comparator reagents to suppress the steady-state level of superoxide or peroxide was interrogated. Finally, using cyclic voltammetry, we demonstrated that, unique among these three phenolic compounds, ATH434 exhibited a chemically reversible electrode electron transfer with a half-wave potential, *E*_1/2_ = 328.5 mV. Our results demonstrate that ATH434 has inherent antioxidant activity, which can support neuroprotection independent of its iron binding. Thus, ATH434 appears unique among small molecules in current clinical trials for the treatment of neurodegenerative disorders characterized by iron overload coincident with oxidative stress. The reagents and metabolic assessments used in the studies described herein are summarized in Figure 1.

**Figure 1:**
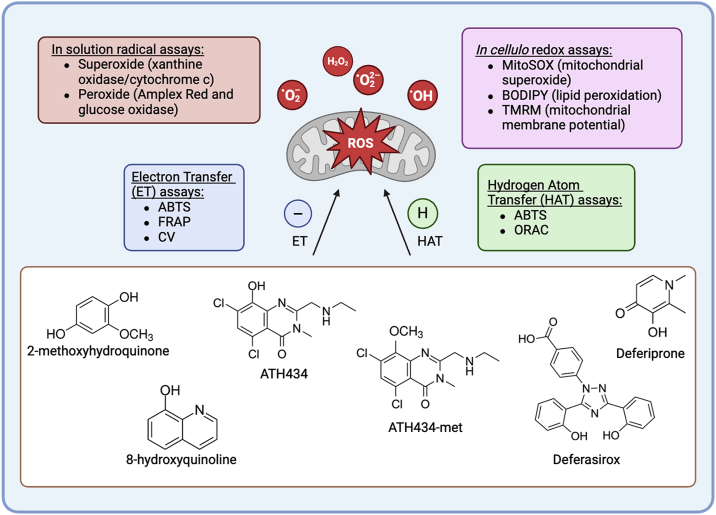
**The structures of the compounds evaluated for chemical and cellular antioxidant activity.** Antioxidant activity was assessed specifically for electron transfer (ET), hydrogen atom transfer (HAT), or both.

## Results

### ATH434 protects HT22 cells from mitochondrial injury

**Figure S1:**
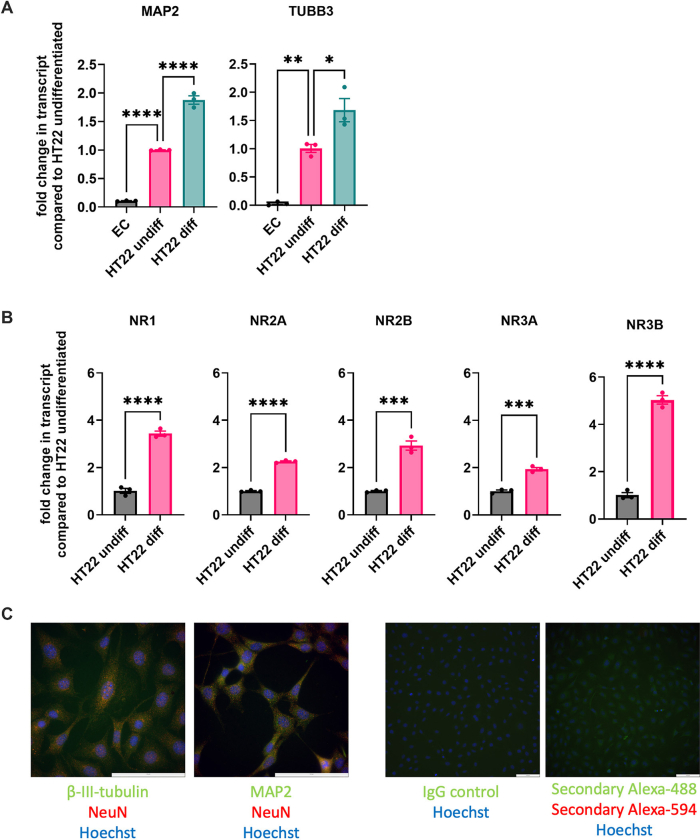


HT22 cells, derived from the murine hippocampus, have been widely used as a model of a glutamatergic neuron (47, 48, 49). Although not commonly noted, its neuronal phenotype requires the use of a differentiation protocol (Methods) involving culture in Neurobasal media containing N2 supplement (50, 51, 52). Expression of neuronal phenotype is demonstrated by both qPCR and protein staining for mature neuronal markers (Fig. S1). With this protocol, the HT22 cells used in all included experiments expressed MAP2 (microtubule-associated protein 2), neuron-specific β-III-tubulin, NMDA receptor subunits as well as glutamate reuptake transporters.

**Figure 2:**
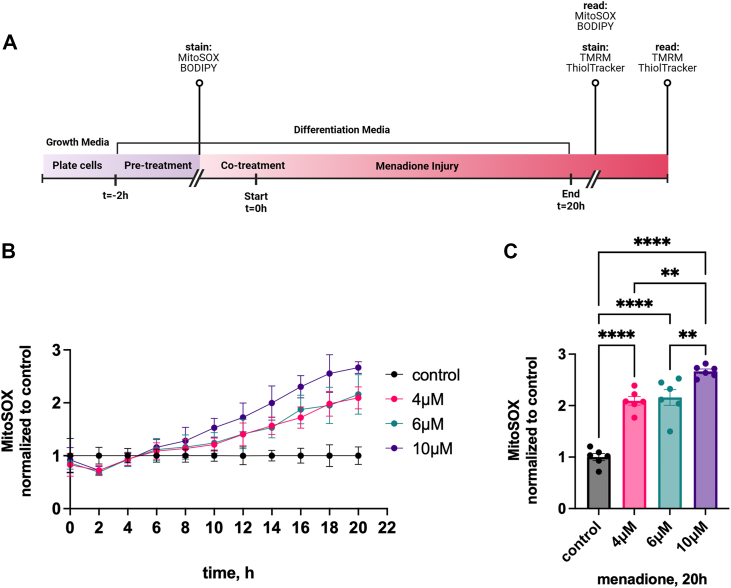
**Optimization of neuronal menadione injury model.***A*, timeline of neuronal menadione injury experiments. HT22 cells were plated on poly-D-lysine coated plates in growth media, then switched to Neurobasal + 1X N2 and 2 mM L- Gln to differentiate for 24 h. For pre-treatment, cells were treated with compound for 2 h prior to the start of the menadione challenge. Cell culture was continued in the absence or presence of menadione for an additional 20 h. Staining was performed prior to menadione treatment or at the end of the assay, as indicated. *B*, dose–response curve for menadione injury in HT22 cells. Cells were stained with MitoSOX, then treated in the absence or presence of increasing concentrations of menadione. Mitochondrial superoxide accumulation was measured every 2 h for 20 h with values expressed relative to MitoTracker *Green* and Hoechst staining, then normalized to untreated control at each timepoint. *C*, mitochondrial superoxide after 20 h menadione injury. MitoSOX signal was normalized to the untreated control. Statistical significance was measured using one-way ANOVA with Tukey’s multiple comparisons test across all treatment groups; ∗, *p* < 0.05; ∗∗, *p* < 0.01; ∗∗∗, *p* < 0.001; ∗∗∗∗, *p* < 0.0001.

To establish an efficacious HT22 oxidant injury model, we investigated the effects of menadione on mitochondrial function. For these assays, cells were plated and allowed to reach 70% confluency and then differentiated for 24 h prior to treatments according to the timeline in Figure 2*A*. Recent work has demonstrated that menadione induced high levels of superoxide and peroxide in cells (53). To assess mitochondrial oxidative stress, we pre-stained cells with MitoSOX to quantify accumulation of mitochondrial superoxide in response to various concentrations of menadione (Fig. 2*B*). Cells were treated for 20 h; MitoSOX fluorescence was quantified every 2 h. We saw a dose-dependent increase in fluorescence over the 20 h treatment at all menadione concentrations. Both 4 and 6 μM menadione induced a 2-fold increase in mitochondrial superoxide compared to control cells; menadione at 10 μM caused a 2.5-fold increase (Fig. 2*C*). In subsequent assays, we chose 6 μM menadione treatment for a maximum of 20 h to induce a statistically significant injury while limiting the potential for cell death (Fig. S2).

**Figure 3:**
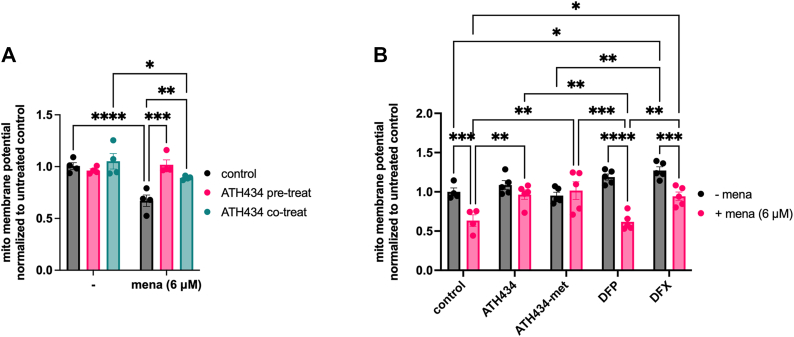
**ATH434 and ATH434-met rescue menadione-induced loss of mitochondrial membrane potential in HT22 cells.***A*, comparison of pre-treatment with ATH434 *versus* co-treatment of ATH434 only on mitochondrial membrane potential (MMP). Menadione injury was induced in the absence or presence of ATH434 co-treatment or pre-treatment with ATH434. MMP was assessed using TMRM assay. Statistical significance was measured using two-way ANOVA with Tukey’s multiple comparisons test across all treatment groups; ∗, *p* < 0.05; ∗∗, *p* < 0.01; ∗∗∗, *p* < 0.001; ∗∗∗∗, *p* < 0.0001. *B*, TMRM assay was used to assess mitochondrial protective effects of test compounds. Conditions: 6 μM menadione for 20 h without or with 20 μM compound pre-treatment for the first 2 h. Data are expressed relative to MitoTracker Green and Hoechst, then normalized to the untreated control. Statistical significance was measured using two-way ANOVA with Tukey’s multiple comparisons test across all treatment groups; ∗, *p* < 0.05; ∗∗, *p* < 0.01; ∗∗∗, *p* < 0.001; ∗∗∗∗, *p* < 0.0001.

We used mitochondrial membrane potential to assess menadione-induced mitochondrial stress. We first demonstrated that ATH434 itself had no effect (Fig. 3*A*). Menadione (6 μM, 20 h) alone induced a 33% decrease in MMP as determined by TMRM staining that was rescued completely with ATH434 (20 μM) pretreatment. ATH434 co-treatment partially rescued the menadione effect (89% of control). In subsequent assays, we used 6 μM menadione with 2 h compound pre-treatment. Using this paradigm, we investigated the activity of ATH434, DFP, and DFX in protection against this menadione-dependent oxidative stress. We also examined the activity of ATH434-met, which lacks ferrous or ferric iron binding activity (18). This structure-function approach allowed for quantification of oxidative stress protection by ATH434 independent of its inherent iron-chelating activity. Cells were incubated in the absence or presence of these structural analogs for 2 h, then incubated in the absence or presence of menadione for another 20 h as above (Fig. 3*B*). We again quantified a 35% decrease in MMP with menadione treatment alone. Significantly, ATH434 and ATH434-met both fully protected cells from menadione-induced loss of MMP, as indicated by a complete rescue back to control levels. In contrast, neither DFP nor DFX suppressed the menadione-induced mitochondrial injury. These findings are consistent with a model in which protection by ATH434 from the redox stress induced by menadione is independent of its activity as an iron chelator; this inference is consistent also with the *lack* of protection afforded by either DFP or DFX. Throughout these studies, we report pharmacologic effect at 20 μM for these small molecules, a choice that reflects a clinically relevant concentration (54).

### ATH434 exhibits inherent antioxidant activity

**Figure 4:**
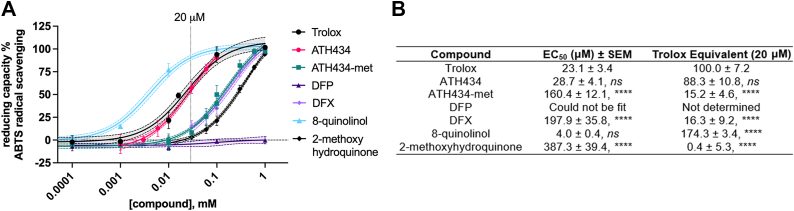
**ATH434 and ATH434-met quench the ABTS radical cation.***A*, the dose-dependent reducing capacity of each compound was determined using the ABTS radical scavenging assay. Trolox was used as a positive control, while 8-quinolinol and 2-methoxyhydruquinone provide structure-activity relationships in ATH434. *B*, data from *panel A* were fit using non-linear regression for dose-response and shown as mean with 95% confidence interval. The EC_50_ ± SEM values for each compound w determined from the non-linear regression fit and statistical significance for EC_50_ values were calculated using one-way ANOVA and Dunnett’s multiple comparisons compared to Trolox; *ns*, *p* > 0.05; ∗∗∗∗, *p* < 0.0001. Data at 20 μM compound were normalized to Trolox; this Trolox equivalent (TE) of antioxidant activity is provided. Statistical significance for TE was calculated using one-way ANOVA and Dunnett’s multiple comparisons compared to Trolox; *ns*, *p* > 0.05; ∗∗∗∗, *p* < 0.0001.

We next examined whether ATH434 and/or the other candidate compounds exhibited antioxidant activity. Most antioxidants can be classified through simple, chemical-based in-solution assays as an initial step in their characterization (55, 56, 57). We first assessed the test compounds using the 2,2′-azino-bis(3-ethylbenzothiazoline-6-sulfonic acid (ABTS) radical assay; this assay screens for both electron transfer (ET) and hydrogen atom transfer (HAT) activity (58). Dose–response curves were generated for ATH434, ATH434-met, DFP, and DFX over a wide range of concentration and compared to Trolox, a Vitamin E analog commonly used as a positive control in these assays (Fig. 4*A*).

In another experiment using the ABTS assay, we tested two analogs of ATH434, 8-quinolinol (8-hydroxyquinoline, 8Q) and 2-methoxyhydroquinone (2-met) (structures shown in Fig. 1), to assess whether there were any structure-activity relationships that coincided with detected antioxidant activity. Based on the dose-response curve, an EC_50_ value of 23.1 μM ± 3.4 μM was calculated for Trolox (Fig. 4*B*). Similar to Trolox, ATH434 had potent antioxidant activity with a calculated EC_50_ value of 28.7 μM ± 4.1 μM. DFX and ATH434-met also exhibited activity, although the EC_50_ values for both were approximately an order of magnitude higher than for Trolox and ATH434 (DFX, 197.9 μM ± 35.8 μM; ATH434-met, 160.4 μM ± 12.1 μM). Notably, DFP exhibited *no* detectable ABTS reducing activity across the tested concentrations up to 1 mM. The 8Q and 2-met structural analogs were positive for ABTS reducing activity as well, with 8Q having the most potent activity out of the compounds included.

The EC_50_ values for compounds positive for activity in Figure 4*A* were compared against ATH434 and the resulting *p*-values are reported in Figure 4*B*. We also calculated the Trolox equivalent (TE) for each compound at 20 μM (dashed vertical line in Fig. 4*A*), corresponding to the reagent concentration used in cell-based assays. ATH434 has a TE value of 88%, a value not statistically different from Trolox. This finding was consistent with ATH434 possessing an *in cellulo* mitochondrial protective effect. With the exception of 8Q, all other compounds had a TE value of ∼15% or less at 20 μM, indicative of a relatively low potency for reducing ABTS when at a physiologically relevant concentration.

### ATH434 exhibits superoxide radical scavenging activity

**Figure 5:**
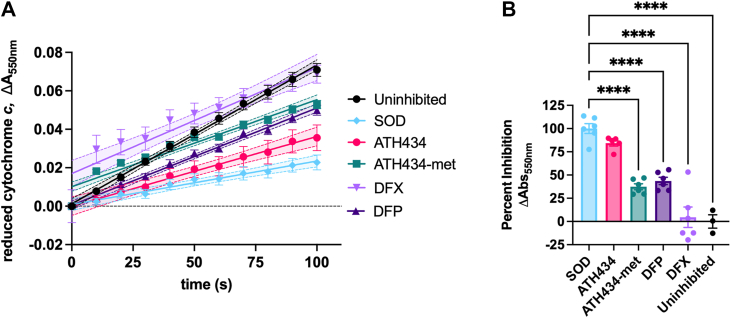
**ATH434 and ATH434-met scavenge superoxide.***A*, superoxide was generated by the xanthine/xanthine oxidase reaction with the absorbance of superoxide-reduced cytochrome c (550 nm) used to quantify the steady-state superoxide level. Compounds (20 μM) were added to the reaction mix prior to the addition of xanthine. The absorbance data were fit using a linear regression analysis and shown as a mean with the 95% confidence interval. *B*, reaction rates were calculated as the slope of the linear regression fits in *panelA*. Statistical significance was calculated compared to SOD; ∗, *p* < 0.05; ∗∗∗∗, *p* < 0.0001.

Scavenging of superoxide was assessed using the classic in-solution assay in which superoxide was generated using xanthine oxidase, and its presence in solution was detected using the increase in absorbance upon reduction of ferricytochrome c (59). Activity was compared relative to the uninhibited reaction as well as to superoxide dismutase (SOD) as a positive control (Fig. 5*A*). These data, expressed as the percent inhibition of the ferricytochrome *c* reduction control, are quantified in Figure 5*B*. SOD inhibited the ferricytochrome *c* reduction (at 100 s) by 100 ± 5.3% while ATH434- and ATH434-met inhibited this reaction by 84.4 ± 3.2% and 37.4 ± 3.2%, respectively. DFP inhibited ferricytochrome *c* reduction by 43.6 ± 3.9%. Ferricytochrome *c* reduction in the presence of DFX was not different from the uninhibited control. Note that superoxide anion scavenging can occur by either electron or hydrogen atom transfer (60).

### ATH434 does not have peroxide scavenging activity

**Figure 6:**
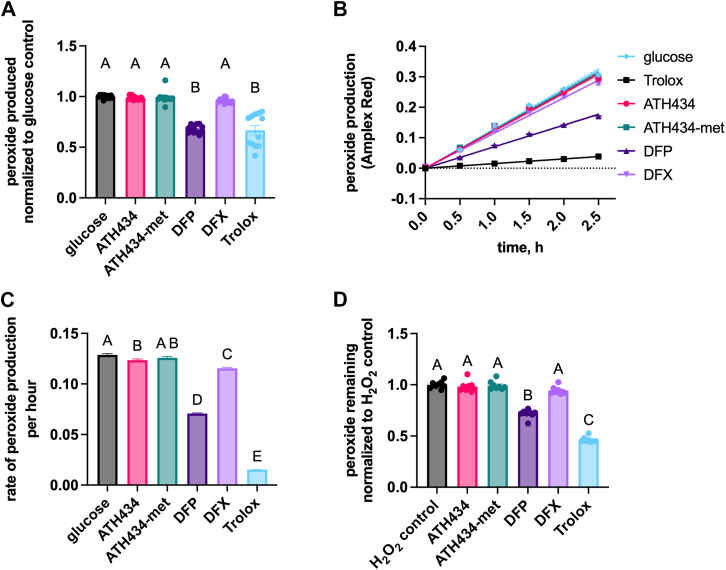
**ATH434 does not scavenge peroxide.***A*, peroxide was continually generated by glucose/glucose oxidase with quantification using an Amplex Red/horseradish peroxidase coupled reaction. Compounds (20 μM) were added to the reaction mix prior to addition of the substrate, glucose. Data were expressed relative to glucose only control and statistical significance was calculated using one-way ANOVA and Tukey’s multiple comparisons test across compounds; groups that are not statistically different (*p* > 0.05) have a shared letter designation. *B*, data collected every 0.5 h up to 2.5 h as in *panelA* were fit using linear regression and shown as mean with 95% confidence interval. *C*, reaction rates were calculated as the slope from the linear regression fits in panel *B*. Statistical significance was determined compared to the glucose only reaction using one-way ANOVA and Tukey’s multiple comparisons test across compounds; groups that are not statistically different (*p* > 0.05) have a shared letter designation *D*, peroxide scavenging by compounds was also calculated using a known starting amount of peroxide. Compounds were incubated with peroxide for 30 min prior to quantification with as above. The amount of peroxide remaining in solution was plotted and statistical significance was determined compared to the peroxide only control using one-way ANOVA and Tukey’s multiple comparisons test across compounds; groups that are not statistically different (*p* > 0.05) have a shared letter designation.

The ability of ATH434 to scavenge hydrogen peroxide was also investigated. Peroxide was generated continuously in solution using the glucose-glucose oxidase reaction. The relative peroxide abundance was assessed by resorufin fluorescence due to the peroxide-dependent horseradish peroxidase turnover of Amplex Red. The amount of peroxide scavenging by our test compounds was assessed compared to the glucose-only control after 2.5 h incubation (Fig. 6*A*). We saw a 67% decrease in the steady-state peroxide level in the presence of Trolox and DFP; ATH434, ATH434-met, and DFX exhibited no comparable activity. We also quantified the rate of peroxide scavenging every 30 min throughout the 2.5 h experiment (Fig. 6*B*). Trolox inhibited the rate of peroxide production by 88% while DFP decreased the rate by ∼45% (Fig. 6*C*). We suspected the effect of DFP could be due to ligation to or chelation of iron out of the horseradish peroxidase heme, similar to the activity exhibited by EDTA (61). To test this inference, we conducted a peroxidase assay using a pre-determined starting concentration of peroxide with pre-incubation of the compounds with peroxide prior to addition of the enzyme (Fig. 6*D*). While there is a brief exposure of the HRP enzyme to DFP at the end of the pre-incubation, we did still see a decrease in detectable peroxide in the presence of DFP (∼30% decline) and Trolox (∼55% decline).

### ATH434 has both HAT and ET activity

**Figure 7:**
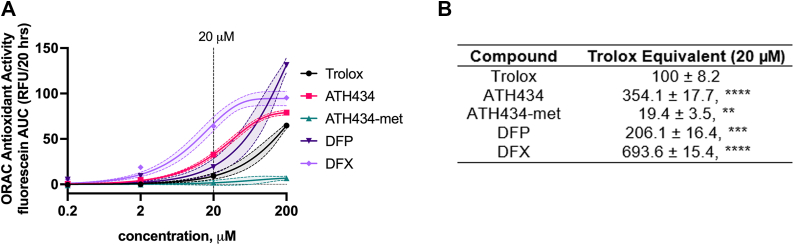
**ATH434 and ATH434-met have HAT activity.***A*, hydrogen atom transfer (HAT) activity was assessed using APPH-derived hydroperoxyl radical quenching of fluorescein fluorescence (ORAC assay). The concentrations used were log transformed with the data fit using linear regression and shown as mean with 95% confidence interval. *B*, data at 20 μM compound were normalized to Trolox providing the Trolox equivalent (TE) reported in the table. Statistical significance was calculated using one-way ANOVA and Dunnett’s multiple comparisons compared to Trolox; ∗∗, *p* < 0.01; ∗∗∗, *p* < 0.001; ∗∗∗∗, *p* < 0.0001.

To more fully understand the ATH434 and ATH434-met structure-function relationship, we expanded our investigation of their anti-oxidant activities using ORAC (Oxygen radical absorbance capacity) and FRAP (ferric reducing antioxidant power) assays. These approaches test the contributions of hydrogen atom transfer (HAT) and electron transfer (ET) to antioxidant activity, respectively. The ORAC assay is based on the quenching of fluorescein by the hydroxyl radical generator 2,2′-azobis(2-amidinopropane) (AAPH). Hydrogen atom transfer from a putative antioxidant inhibits this ROS-induced fluorescein quenching. A range of concentrations centered around the EC_50_ values determined in the ABTS assay (above) was tested, and the area under the curve (AUC) concerning the delay in fluorescein quenching by AAPH-derived radical species was calculated for each. These AUC concentration curves are shown in Figure 7*A*, with the Trolox equivalent (TE) values at 20 μM provided in Figure 7*B*. All except ATH434-met exhibited 2 to 3.5-fold *more* HAT activity than Trolox in this assay. That is, methylation of the phenol in ATH434 abrogated a major fraction of the HAT activity exhibited by its parent compound, indicating that the phenolic hydrogen atom in ATH434 and likely in DFP and DFX as well, contributes to this aspect of observed antioxidant activity. Note, however, that ATH434-met exhibits 20% of the HAT activity of the antioxidant standard, Trolox.

**Figure 8:**
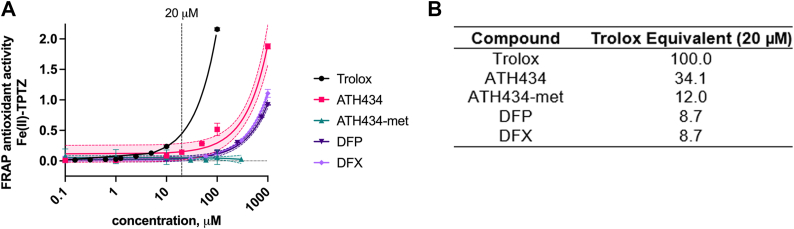
**ATH434 has ET activity while ATH434-met does not.***A*, electron transfer activity was assessed using the FRAP assay. The reduction of ferric 2,4,6-Tris(2-pyridyl)-s-triazine was quantified by the increase in absorbance at 594 nm. Data were fit using linear regression, shown as the mean with a 95% confidence interval. *B*, data at 20 μM were normalized to Trolox, providing the Trolox equivalent (TE) reported in the table. The interpolation from a single concentration value prevents calculation of the SEM for these values.

To assess the contribution of ET to the antioxidant activity of these compounds, we subjected them to a FRAP assay (Ferric Reducing Antioxidant Power) in which outer sphere electron transfer to ferric tris(2-pyridyl)-s-triazine (TPTZ, electrode potential >800 mV) from a putative antioxidant is quantified spectrophotometrically. The reduction of this FRAP reagent as a function of reagent concentration is quantified in Figure 8*A*. The Trolox Equivalent (TE) values at 20 μM were interpolated from the linear regression fit and are reported in Figure 8*B*. Noticeably, ATH434-met exhibited no electron transfer activity, a behavior that correlated with its lower activity than ATH434 in the ABTS assay which reports on both HAT and ET function. This result is consistent with the inference that the phenol moiety in Trolox and the three small molecule therapeutics is the electron donor in this redox reaction. However, the Trolox equivalent FRAP activity found for DFP and DFX was less than 9% that of Trolox highlighting the variable efficacy of ET from the phenol moiety that Trolox, ATH434, DFP, and DFX possess to different electron acceptors.

### ATH434 exhibits a 328.5 mV chemically reversible electrode potential

**Figure 9:**
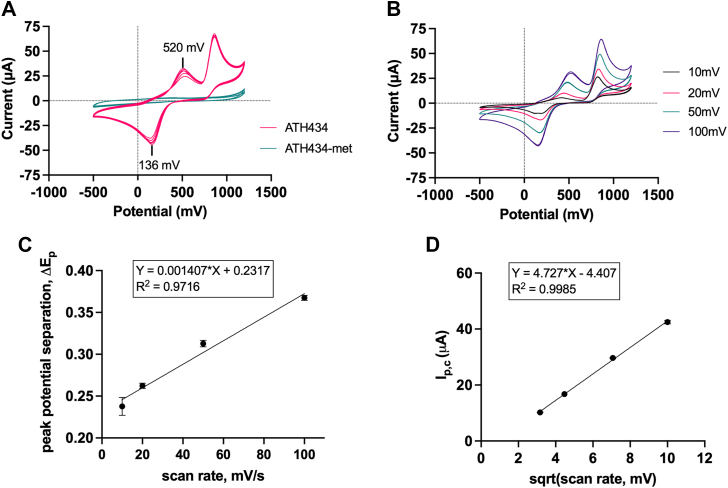
**ATH434 exhibits a chemically reversible one-electron transfer at a carbon electrode while ATH434-met does not.***A*, electron transfer activity of ATH434 (*red trace*) and ATH434-met (*green trace*) was assessed using cyclic voltammetry. Data are shown for n = 3 runs with 2 cycles per run at 100 mV/s. The cathodic and anodic peak potentials are noted on the voltammogram. *B*, voltammograms for ATH434 at decreasing scan rate. Data are shown for n = 2 cycles for each scan rate. Note the expected decrease in both the peak potential separation, ΔEp, and cathodic peak current, Ip,c. *C*, Peak potential separation, ΔEp, was plotted *versus* scan rate. *D*, Cathodic peak current, Ip,c, was plotted *versus* the square root of the scan rate.

The data above demonstrate that ATH434 can support both electron and hydrogen atom transfer to a suitable redox partner. We utilized cyclic voltammetry (CV) to confirm that the electron transfer activity of ATH434 is chemically reversible, and to measure the electrode potential of ATH434 at physiologic pH that likely supports this ET activity (Fig. 9*A*). Two anodic oxidation peaks were observed in the forward cyclovoltammetry scan at 100 mV/s, one at 522.5 mV ± 10.2 mV and the other centered around 890 mV (pink trace). While the latter peak appears to be chemically irreversible as it lacks a complementary reduction wave, we observed a single cathodic reduction peak at 134.4 mV ± 33.2 mV that corresponded to the 520 mV anodic peak. From these data, we calculated a half-wave potential for ATH434 at physiologic pH, *E*_1/2_ = 328.5 mV ± 14.4 mV. The chemically irreversible oxidation peak near 900 mV without a corresponding reduction peak is characteristic of phenols (62, 63). Both DFP and DFX have been reported to exhibit this phenol-like electrochemical behavior as well (64, 65).

The voltammogram for ATH434 compares to the quasi-reversible CV process characteristic of hydroquinone-based compounds (66, 67, 68, 69, 70). Electrode reversibility in CV relates to the relative rates of reagent diffusion to and electron transfer at the electrode, *not* to the chemical reversibility of the oxidation-reduction process occurring at the electrode. To examine in more detail the apparent quasi-reversible behavior for ATH434, scans were recorded as a function of scan rate to probe the electrode kinetics for the ATH434 oxidation-reduction cycle (Fig. 9*B*). The peak potential separation ΔE_p_ was calculated for each scan rate. With decreasing scan rates, the anodic and cathodic midpoint peaks for ATH434 shift closer together as expected, with the calculated ΔE_p_ decreasing linearly (Fig. 9*C*), consistent with an electrode reaction that trends towards kinetic reversibility at slower scan rates (66, 68). Plotting the peak current at the cathode *versus* the square root of the scan rate (Fig. 9*D*) also shows a linear relationship, consistent with such a process. Note, however, that even at the slowest scan rate, electron transfer at the electrode never reached reversibility. Thus, the redox cycle at the electrode fails to attain a true Nernstian equilibrium. However, irrespective of the kinetic nature of the electron transfer itself, the data show that ATH434 undergoes a *chemically* reversible, likely one-electron transfer redox reaction, with a half-wave potential of 328.5 mV at pH 7.4. Independent of that process, this hydroxyquinazolinone exhibits another but chemically irreversible oxidation at the electrode characteristic of phenols.

CV scans were also conducted for ATH434-met to probe the contribution of the 8-hydroxyl group to the electrochemical behavior of ATH434. Indeed, the 890 mV chemically irreversible oxidation peak was absent from the voltammogram for ATH434-met (Fig. 9*A*, green trace), in support of the assignment of this process to the phenol moiety. Notably, methylation of the 8-hydroxyl group led to a loss of one electron, a 329 mV chemically reversible process. Together, the CV data for ATH434 and its methoxy congener suggest that the phenolic -OH is responsible for *both* electrode reactions involving the parent compound.

### Antioxidant activity of ATH434 suppresses menadione-induced lipid peroxidation in HT22 cells

**Figure 10:**
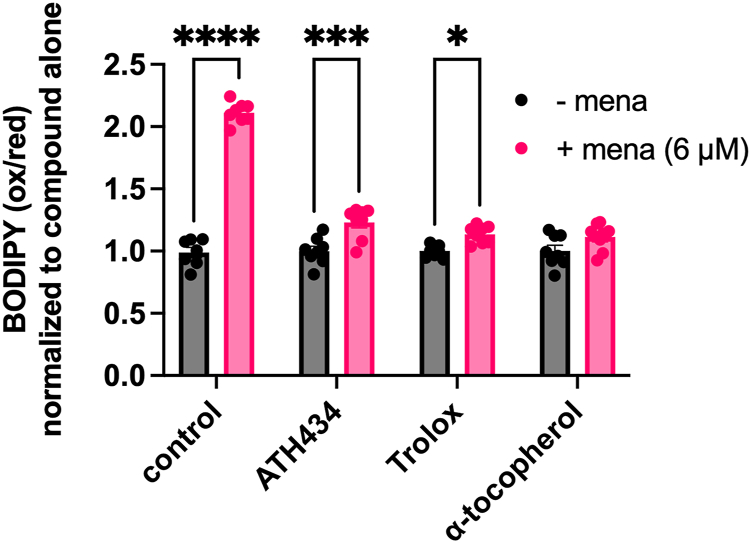
**ATH434 protects HT22 cells from menadione-induced lipid peroxidation.** C11-BODIPY581/591 was used to assess lipid peroxidation in HT22 cells. Cells were pre-treated with the indicated compounds (20 μM) for 2 h, stained with dye, then treated in the absence or presence of menadione (6 μM) in the continued presence of the compound for an additional 20 h. Fluorescence was quantified with data expressed as the ratio of oxidized/reduced reporter. Reagent ox/red ratio was normalized to the non-menadione control for each treatment group. Statistical significance was calculated using two-way ANOVA and Tukey’s multiple comparisons test within treatment groups; ∗, *p* < 0.05; ∗∗∗, *p* < 0.001; ∗∗∗∗, *p* < 0.0001.

To validate that the antioxidant activity exhibited by ATH434 translated to a quantifiable antioxidant effect in cells, we characterized its protective activity in the context of lipid peroxidation induced by menadione. Thus, we treated HT22 cells with ATH434 or antioxidants Trolox or α-tocopherol; the cells were pre-stained with C11-BODIPY 581/591 prior to quantifying the accumulation of lipid peroxidation (Fig. 10). Menadione alone induced a 2.3-fold increase in the BODIPY oxidized/reduced ratio, indicative of increased lipid peroxidation. Pre-treatment with ATH434 decreased the menadione-induced lipid peroxidation ∼90%, a suppression equivalent to that supported by both Trolox and α-tocopherol.

## Discussion

In this study, we investigated the neuroprotective and antioxidant properties of ATH434 in comparison to its non-iron-binding analog ATH434-met and comparator iron chelators DFP and DFX. Most significantly, ATH434 exhibits a chemically reversible one-electron electrode reaction not exhibited by either comparator phenol-containing compounds, including DFP (64) or the commonly used antioxidant standard, Trolox (71). In addition, we found ATH434 has an inherent antioxidant activity similar to Trolox and α-tocopherol. Significantly, this activity is independent of its iron-binding capacity inasmuch as ATH434-met exhibits similar, albeit quantitatively variable, activity in a variety of antioxidant assays.

While other metal chelating small molecules have traditionally been used to bind excess circulating iron in iron overload disorders, they have had limited success in treating the excess iron associated with pathology in neurodegenerative disorders. Thus, DFP and DFX, with pFe^3+^ values of 20 or more, can sequester iron out of metabolically essential enzymes and proteins, an attribute that has limited their usefulness (24). Indeed, we saw evidence of this in our study, with exacerbation of menadione effects on mitochondrial function and cellular redox balance with DFP and DFX. DFP also gave evidence of HRP inhibition in the peroxide assay. This inhibition of HRP enzyme activity could be analogous to the effect of DFP on tyrosine hydroxylase, a mono-nuclear iron enzyme (24), *i.e.,* the chelation of Fe out of the protein leading to a loss of function. While there was an apparent peroxide scavenging capacity of DFP, this effect could not be separated from an impact on HRP itself.

A recent study (18) compared the Fe^2+^ binding affinities of ATH434, ATH434-met, DFP, and DFX using spectroscopic metal competition and isothermal titration calorimetry (ITC) techniques. The study confirmed the *moderate* iron-binding affinity of ATH434, with a measured average *K*_d_ value of 4.85 ± 1.8 μM for Fe^2+^ at pH 7.5 and 0.46 ± 0.04 μM for Fe^3+^ at pH 5.5. This study also demonstrated a similar DFX Fe^2+^ dissociation constant (4.25 ± 1.3 μM); the stability of the DFP•Fe^2+^ was weaker by a factor of 3. The most significant difference between ATH434 on the one hand, and DFP and DFX on the other is the ∼10^14^-fold greater stability of the ferric iron complexes of the latter two. Their profoundly greater Fe^3+^ complex stabilities in comparison to Fe^2+^ support their effective potentiation of the autooxidation of Fe^2+^ in aerobic environments. Rather than decreasing oxidative stress *via* iron chelation, DFP and DFX are likely to contribute to radical generation (20, 21). Given its quantitatively similar and moderate affinity for *both* Fe^2+^ and Fe^3+^, *thermodynamically,* ATH434 poses a more limited iatrogenic challenge to cell redox and iron balance.

The key finding in this study was the antioxidant activity of ATH434 and of ATH434-met in some assays. This is indicative of inherent ET and/or HAT activity in the structure of ATH434 and ATH434-met. The variable activity in assays differing in chemistry provides a basis for understanding the structure-activity relationships in this 8-hydroxy-3-methylquinazolin-4(3H)-one. First, we can attribute the ET antioxidant activity of ATH434 to its 8-hydroxy/phenol moiety since that activity is attenuated if not lost in ATH434-met. This inference is supported by the activity of the structural analog 8-hydroxyquinoline, which was the most efficacious of all compounds tested in the ABTS assay. This activity is contributed by its phenolic oxygen, perhaps potentiated by resonance involving the pyrimidine nitrogen. Similar hydroxyquinoline-based structures have been used as the basis for the development of therapeutics such as clioquinol (CQ) and a hydroxyquinoline compound, PBT2; the former has been successful in targeting iron overload in the substantia nigra in non-human primate PD models (72) and the latter in targeting amyloid-beta in AD mouse models (73, 74). While our data support the utility of 8-hydroxyquinoline as an antioxidant, this latter compound and its derivatives have had limited efficacy *in vivo* due to their lower blood-brain barrier permeability (75, 76) compared to second-generation hydroxyquinoline derivatives like PBT2 (74, 77, 78). ATH434 readily crosses the BBB both *in vitro* (46) and *in vivo* (54), a feature that likely contributes to success in its use in animal models of neurodegenerative disease and apparent efficacy in managing the progression of MSA in patient trials.

Surprisingly, ATH434-met was as effective as ATH434 in support of the MMP even though its EC_50_ values were 3 to 15-fold higher than for ATH434 in the various *in vitro* assays. This reduced antioxidant efficacy could be related to the loss of ET potential in ATH434-met due to the methylation of the phenol moiety. On the other hand, retention of HAT activity in the ORAC assay could explain why ATH434-met retains some efficacy in cellular reduction of menadione-induced oxidative stress. Based on the shared aspects of the structure, we hypothesize that a benzylic hydrogen atom(s) of the 2-(ethylamino)methyl sidechain could be the source of a hydrogen atom contributing to this HAT activity. Density functional theory studies have demonstrated that benzylic protons like those in ATH434 play a significant role in antioxidant function (79).

As an important complement to in-solution assays, we directly assessed the redox potential of ATH434 using cyclic voltammetry and compared its behavior at the electrode to that of ATH434-met. The half-wave potential for ATH434 was found to be 328.5 mV ± 14.4 mV at pH 7.4, which poises ATH434 for reversible one-electron oxidation-reduction within the cellular environment, between CoQ (100 mV) and Vitamin E (370 mV), and comparable to Cyt *c*1 (220 mV) in terms of effective reduction potential. In contrast, DFP and DFX exhibit only a chemically irreversible anodic oxidation at ∼900 mV common to all phenolic compounds (64, 80). Indeed, ATH434 also exhibits this feature as indicated by a comparable chemically irreversible electrode reaction.

Of note is that the 329 mV reduction potential exhibited by ATH434 electrochemically places it among *all* superoxide dismutases irrespective of the latter’s redox-active cofactor (81). The Cu^2+^/Cu^1+^ potential in bovine superoxide dismutase, for example, is 403 mV (82). A reduction potential in this range for the metal places it at the mid-point between the −350 mV potential of the O_2_/O_2_^−•^ couple and +910 mV potential of the O_2_^−•^/H_2_O_2_ one (32). However, our superoxide scavenging assay *does not* demonstrate a *dismutation* activity for ATH434, only that this quinazolinone derivative has the capacity to suppress superoxide steady-state level as generated by the xanthine-xanthine oxidase reaction. In this assay, the superoxide radical could oxidize ATH434 by one electron without subsequent cycling of the ATH434 radical cation in conversion of a second O_2_^−•^ to dioxygen. Interrogation of the redox chemistry of ATH434 in this regard was beyond the scope of this report.

As noted earlier, however, superoxide can also be quenched by transfer of a hydrogen atom, HAT (60). This fact offers a rationale for the superoxide quenching activity retained by ATH434-met despite its lack of ET activity in cyclic voltammetry and its more limited activity in various antioxidant assays. Indeed, the H-atom transfer potential that appears to be characteristic of both hydroxyquinazolinone derivatives is likely a component of their suppression of the oxidative stress elicited by menadione in HT22 cells. Menadione is an efficient generator of cellular superoxide (53).

A 20 μM concentration of ATH434 was utilized in these studies based on relevance to corresponding levels found in the CSF (54); this concentration was used for all compounds in our experimental design here and has been used previously (46). The apparent antioxidant activity of ATH434, as indicated by interpolated EC_50_ values, varied. Thus, the EC_50_ for ATH434 in the quenching of the ABTS ^+^ radical was not significantly different from that of Trolox (28.7 ± 4.1 μM *versus* 23.1 ± 3.4 μM, respectively). This behavior correlated with the *in cellulo* activity for ATH434 in mitochondrial protection and for ATH434, Trolox, and α-tocopherol in protection from lipid peroxidation. On the other hand, our data show that ATH434 has a Trolox equivalent of 354.1% for HAT activity but only 34.1% for ET activity. The apparent low potency of ATH434 in ET may reflect the fundamentally different chemistries of ET *versus* HAT-based reactions in terms of thermodynamics. According to Marcus theory, the rate of an outer sphere ET reaction is, in part, driven by its thermodynamic driving force (83). Inasmuch as ET into the ferric iron in the TPTZ ferric iron complex is outer sphere, the coupling of the electron donor (the antioxidant) to the Fe^3+^ will be dependent also on orbital energy factors that may or may not have any relationship to how the small molecule might serve an antioxidant function in a cell. Similarly, the different ET assays differ with respect to the reduction potential of the reporter used: DPPH^+^ (400 mV) *versus* ABTS^+^ (500 mV) *versus* TPTZ-Fe^3+^ (770 mV) (84, 85, 86). As the reduction potential of the reporter increases, thermodynamically the activity reported does too, albeit modulated by the other Marcus factors that predict ET rates (83, 86). Of course, factors such as cell penetrance and distribution further discourage aggressive correlation between test-tube and *in cellulo* antioxidant measurements. In summary, we take antioxidant activity to be a *behavior* rather than a quantity, particularly when it comes to a small molecule’s efficacy in suppressing some measure of cellular oxidative stress.

In this study, we used a superoxide and peroxide generator, menadione, to induce oxidative stress in our HT22 cell neuronal model to assess the effects of ATH434 and comparator compounds. This initial screening of activity and efficacy sets up ATH434 as a potential therapeutic in disorders characterized by oxidative stress and iron overload. Importantly, the moderate iron binding of a compound like ATH34 could help to chaperone iron to subcellular locations or proteins that are iron-deficient. Concurrent antioxidant activity could prevent or rescue the oxidative stress generated by the redox cycling of oxygen fueled by iron directly or other metabolic pathophysiology.

## Experimental procedures

### Reagents

ATH434 and ATH434-met were provided by Alterity Therapeutics (Melbourne, AUS). DFP (Cat. #HY-B0568), DFX (Cat. #HY-17359), 8-Quinolinol (Cat. #HY-B1005) were purchased from MedChemExpress. Trolox (Cat. #10011659) and a-tocopherol (Cat. #25985) were purchased from Cayman Chemical. N-ethylmaleimide (Cat. #E1271), 2-methoxyhydroquinone (Cat. #176893), and menadione (Cat. #M5625) were purchased from Sigma (Sigma-Aldrich). All compounds were reconstituted in DMSO. Live-cell imaging solution was made according to Life Technologies (Thermo) with 140 mM NaCl, 2.5 mM KCl, 1.8 mM CaCl_2_, 1.0 mM MgCl_2_, and 20 mM Hepes, pH 7.4.

### Cell culture

HT22 mouse hippocampal neuronal cells were obtained from EMD Millipore (Millipore Sigma). Cells were grown in DMEM (Cat. #11965, Thermo Fisher Scientific) with 10% FBS (Cat. # A5256701, Thermo), 2 mM L-Glutamine (Cat. #25-005-CI, Corning), and 1X pen/strep (Cat. #30-002-CI, Corning). For experiments, cells were plated on poly-D-lysine (PDL, Cat #A3890401, Thermo) coated tissue culture plates. Cells were grown to 60 to 70% confluency, then differentiated with Neurobasal media (Cat. #21103049, Thermo) containing 1X N2 supplement (Cat. #17502001, Thermo), 2 mM L-Gln for 24 h prior to assay (51, 87).

### qPCR

HT22 cells were grown in PDL-coated 6-well plates in either growth media (undiff) or differentiation media (diff) for 24 h as described above. Cells were washed twice with phosphate-buffered saline (PBS), and total RNA was isolated using TRIzol reagent (Invitrogen). RNA was purified using the Zymo Direct-zol RNA kit (Zymo Research), and 400 ng of RNA was reverse-transcribed using the qScript cDNA synthesis kit (Quanta Bio) to generate cDNA. Real-time quantitative qPCR was performed using 20 ng of cDNA with the iTaq Universal SYBR Green Supermix and analyzed using a CFX96 Touch real-time PCR detection system (Bio-Rad). The amplification data were normalized to the mean β-actin Ct as the housekeeping gene, and the fold change in transcript level was calculated using the 2ˆ-ΔΔCt method compared to undifferentiated HT22 cells. The primer sequences used are reported in Table S1. Human brain microvascular endothelial cells (EC) cultured as previously described (46) were used as a non-neuronal cell control.

### Immunocytochemistry and Microscopy

HT22 cells were plated on PDL-coated coverslips and differentiated for 24 h as described above. Cells were fixed using 3.7% paraformaldehyde in PBS containing 1.8 mM CaCl_2_ and 0.5 mM MgCl_2_, blocked and permeabilized with 1% BSA with 0.3 M glycine and 0.1% Tween-20 in PBS, then incubated overnight at 4 °C with primary antibody at 1:1000 in 1% BSA as follows: mouse anti-MAP2 (Cat #NBP2-25156, Novus), rabbit anti-NeuN (Cat #NBP1-77686, Novus), mouse anti- β-III-tubulin (Cat #MAB1195, Biotechne), mouse IgG2A isotype control (Cat #MAB003, Biotechne). Coverslips were then incubated with secondary antibody at 1:1000 in 1% BSA with donkey anti-mouse Alexa-488 (Cat #R37114, Invitrogen) or donkey anti-rabbit Alexa-594 (Cat #R37119, Invitrogen), as indicated for 1 h at room temperature, then stained with 0.7 μg/ml Hoechst 33342 as a nuclear marker for 10 min at 37 °C. After washing, coverslips were mounted on glass microscope slides using ProLong Glass Antifade mounting media (Invitrogen). Images were obtained on a Leica DMi8 inverted microscope using either a 20X objective or a 63X objective with oil immersion. Images were adjusted for brightness and contrast using ImageJ.

### TMRM assay

TMRM was purchased from Invitrogen (Cat. #T668, Thermo) and reconstituted in DMSO at 5 mg/ml according to the manufacturer’s instructions. MitoTracker was purchased from Invitrogen (Cat. #M7514, Thermo) and reconstituted at 1 mM in DMSO according to the manufacturer’s instructions. Hoechst 33342 (Invitrogen) was used as a nuclear stain. TMRM staining solution was as follows: 10 μg/ml TMRM, 100 nM MitoTracker Green, and 0.7 μg/ml Hoechst 33342 in live-cell imaging solution. Cells were plated in 24-well or 96-well PDL-coated plates, then differentiated for 24 h prior to assay. Cells were either pre-treated for 2 h with the indicated compounds or co-treated with menadione in the absence or presence of compounds for an additional 20 h. After treatment, cells were washed twice with live-cell imaging solution, incubated with TMRM staining mix for 30 min at 37 °C, washed twice, and fluorescence was read on a plate reader (FLUOStar Omega, BMG Labtech) at the following wavelengths: TMRM ex/em 544/620 nm, MitoTracker Green ex/em 480/520 nm, Hoechst ex/em 355/460 nm. The TMRM signal was normalized to MitoTracker Green and Hoechst signal for the number of mitochondria per cell, then expressed relative to the untreated control.

### MitoSOX assay

MitoSOX Red was purchased from Invitrogen (Cat. #M36008, Thermo) and reconstituted at 5 mM according to the manufacturer’s instructions. MitoSOX staining mix was as follows: 500 nM MitoSOX, 100 nM MitoTracker Green, and 0.7 μg/ml Hoechst in live-cell imaging solution. Cells were plated on PDL-coated 24-well or 96-well culture plates, then differentiated for 24 h prior to assay. Cells were either pre-treated for 2 h with the indicated compounds or co-treated with menadione in the absence or presence of compounds for an additional 20 h. After treatment, cells were washed twice with live-cell imaging solution, incubated with MitoSOX staining mix for 30 min at 37 °C, washed twice, and fluorescence at ex/em 396/610 nm was read on a Cytation 5 plate reader (Agilent BioTek). MitoSOX signal was normalized to MitoTracker Green and Hoechst signal for the number of mitochondria per cell, then expressed relative to the non-menadione control for each compound treatment group.

### ABTS assay

ABTS (2,2′-Azino-bis(3-ethylbenzothiazoline-6-sulfonic acid) diammonium salt; Cat # A1888) and potassium persulfate (Cat # 216224) were purchased from Sigma-Aldrich. ABTS (7 mM) was incubated with potassium persulfate (2.5 mM) in the dark at room temperature for 16 h to generate the ABTS·^+^ radical cation. ABTS working solution was made by diluting ABTS·^+^ in 95% ethanol, then added to wells of a 96-well plate containing compounds at indicated concentrations, n = 6 replicates per concentration. The reaction was allowed to proceed for 90 min in the dark, then absorbance of ABTS at 734 nm was measured on a plate reader. Each compound was first assayed using a wide, log-scale concentration range and compounds positive for ABTS reducing activity were further characterized using a narrow concentration range centered around the effective EC_50_ in the initial run. Data were blank corrected, then normalized as a percentage of the ABTS positive control and expressed as mean ± SEM. Dose-response curves were plotted on a log scale, fit using non-linear regression, and the resulting EC_50_ values were reported.

### Superoxide scavenging assay

Superoxide scavenging was assessed in solution using a xanthine oxidase/cytochrome *c* coupled reaction (88). Cytochrome c (Cat #C2037), xanthine (Cat #X0626) and xanthine oxidase (Cat #X1875) were purchased from Sigma-Aldrich. A reaction mix was made using 10 Units/ml xanthine oxidase and 1.1 mM ferricytochrome *c* in 216 mM potassium phosphate buffer, pH 7.8 containing 10.7 mM EDTA. Bovine superoxide dismutase (Cat #S5395; Sigma-Adrich) at 20 Units/ml was used a positive control for superoxide scavenging. Compounds were added to the reaction mix at 20 μM concentration. Xanthine (0.108 mM) was added to initiate the reaction. Reaction kinetics were monitored by measuring the absorbance of the reactions at 550 nm using a plate reader, with scans every 10 s for a total of 300 s to allow reactions to reach equilibrium. Data were blank-corrected and plotted for the initial, linear phase of the reaction. Data were fit using non-linear regression to calculate the reaction rates (AU/s), and rates were compared across compounds.

### Peroxide scavenging assay

Peroxide scavenging was assessed for continuous peroxide generation using the Amplex Red Glucose/Glucose Oxidase Assay kit (Cat # A22189; Thermo Fisher Scientific). A reaction mix was made, including glucose, glucose oxidase, HRP, and Amplex Red in 1X reaction buffer. Compounds were added to the reaction mix at 20 μM, n = 6 replicates. Absorbance at 560 nm was measured using a plate reader every 30 min for 2.5 h total. Data at each time point was normalized to the glucose-only control and fit using linear regression to determine the rate of peroxide scavenging and peroxide remaining in solution at the end of the assay. Peroxide scavenging was also assessed using a constant concentration of peroxide. A reaction mix was made using 100 mM Amplex Red and 0.2 Units/ml HRP in 50 mM sodium phosphate buffer, pH 7.4. Peroxide (100 mM) was pre-incubated with 20 μM compound for 30 min, then an equal volume of reaction mix was added to measure peroxide remaining in solution. Absorbance at 560 nm was measured using a plate reader. Data was normalized to the peroxide only control and compared across treatments.

### ORAC assay

Oxygen radical absorbance capacity (ORAC) assay was used to assess the hydrogen atom transfer antioxidant activity. ORAC assay utilizes quenching of fluorescein as a reporter for radical production, with delayed quenching indicative of antioxidant capacity. AAPH ((2,2′-Azobis(2-methylpropionamidine) dihydrochloride; Cat # 440914) and fluorescein (Cat # 46955) were purchased from Sigma-Aldrich. AAPH solution was prepared in 75 mM phosphate buffer pH 7.4 at 75 mM final concentration, made fresh. Fluorescein stock solution was made in 75 mM phosphate buffer at 4 μM final concentration. Before use, fluorescein stock was diluted to 80 nM in 75 mM phosphate buffer. 96 well plates were loaded with 150 μl fluorescein working solution and 25 μl test compounds at various concentrations (0.2–200 μM) and incubated at 37 °C for 10 min. The reaction was initiated with 25 μl AAPH solution. Fluorescence measurements were taken every 120 s for 120 min total at ex/em 485/528 nm. Area under the curve (AUC) was calculated for each test compound and blank corrected to determine the net AUC. ORAC activity standard curves were determined by plotting the net AUC for each concentration tested and fit using linear regression. Trolox equivalent response for each test compound at 20 μM was reported.

### FRAP assay

The Ferric-Reducing Antioxidant Power (FRAP) assay was used to quantify the electron transfer activity. FRAP antioxidant activity is quantified by measuring the change in absorbance at 593 nm of an Fe(III)-TPTZ complex upon reduction by antioxidants. TPTZ (2,4,6-Tris(2-pyridyl)-*s*-triazine; Cat # 93285) was purchased from Sigma-Aldrich. Ferric chloride (Cat #I88) was purchased from Fisher Scientific. An assay reaction mix was made fresh with 10 parts (by volume) of 300 mM acetate buffer pH 3.6, 1 part TPTZ and 1 part ferric chloride (each 20 mM). 96-well plates were loaded with 280 μl of FRAP assay mix and 20 μl of sample. The plate was gently shaken to mix and incubated at 37 °C in the dark for 30 min, with all reactions allowed to reach completion. Absorbance was read at 593 nm. Various concentrations of compounds (0.1 μM–1000 μM) were assayed to generate dose-response curves and results were fit using linear regression. Trolox equivalent response for each test compound at 20 μM were interpolated from the best-fit curves; therefore, no SD or SEM was calculated.

### Cyclic voltammetry

Cyclic voltammetry was performed using a WaveNow^XV^ potentiostat and analyzed using Aftermath software (Pine Research Instrumentation, Inc). Compounds were prepared as 5 mM solutions in 0.1 M KCl, 10% DMSO, pH 7.4, then bubbled with nitrogen for 5 to 10 min prior to measurement. Currents for each sample were recorded at a glassy carbon disk electrode with a 100 mV/s scan rate from −500 mV to 1200 mV under ambient atmosphere at 25 °C. Samples were measured against a Ag/AgCl reference electrode, 6 segments per scan, n = 3 scans per compound. Data were also collected at different scan rates (10–100 mV/s). These data were plotted as peak potential separation, ΔEp, *versus* scan rate (mV/s) and cathodic peak current, I_p,c_ (mA) *versus* potential (mV) to assess kinetic reversibility of electron transfer at the electrode.

### Lipid peroxidation

The Image-iT Lipid peroxidation kit was purchased from Invitrogen (Cat. #C10445, Thermo). BODIPY staining mix was as follows: 10 μM C11-BODIPY 581/591 reagent and 0.7 μg/ml Hoechst in live-cell imaging solution. Cells were plated in 24-well or 96-well PDL coated plates, then differentiated for 24 h prior to assay. Cells were either pre-treated for 2 h with indicated compounds or co-treated with menadione in the absence or presence of compounds for an additional 20 h. After treatment, cells were washed twice with live-cell imaging solution, incubated with BODIPY staining mix for 30 min at 37 °C, washed twice, and fluorescence was read on a plate reader (FLUOStar Omega, BMG Labtech). Oxidized BODIPY fluorescence was measured at ex/em 488/510 nm and reduced BODIPY was measured at ex/em 581/591 nm. Lipid peroxidation was expressed as oxidized BODIPY/reduced BODIPY signal, then expressed relative to non-menadione control for each compound treatment group. Hoechst staining was used to confirm equivalent cell number across treatments.

### Statistical analysis

All statistical analyses were performed using Prism 10 (GraphPad Prism software). Data are presented as scatter plots showing all data points, along with a bar representing the mean ± SEM, and n is equivalent to the number of biological replicates for each condition unless otherwise noted. Comparisons between two conditions (one variable) were made using unpaired *t* test. Comparisons between multiple samples were made using one-way ANOVA statistical analyses. For comparison of samples between 2 groups (two variables), two-way ANOVA statistical analysis was used. For all statistical analyses, ns, not significant; ∗, *p* < 0.05; ∗∗, *p* < 0.01; ∗∗∗, *p* < 0.001; ∗∗∗∗, *p* < 0.0001.

## Data availability

All relevant data are contained within this article and the Supporting Information.

## Supporting information

This article contains Supporting Information.

## Conflict of interest

The authors declare the following financial interests/personal relationships which may be considered as potential competing interests: MJB and SB are employed by Alterity Therapeutics; AT has provided partial funding for this research including salary support for DKB and RN. DJK declares that he has no conflict of interest with respect to the research described in this article. The Research Agreement between Alterity Therapeutics and the State University of New York at Buffalo contains no NDA nor restrictions on scientific data release, sharing or publication. DKB, RN and DJK are employees of the State University of New York at Buffalo (DJK) and the Research Foundation of the State University of New York (DKB, RN) who have no competing interest in this research. DKB serves as a consultant for Alterity Therapeutics.

## Figures and Tables

**Figure S2:**