# Supplementary material for: Potent antioxidant and mitochondrial-protective effects of ATH434, a moderate affinity iron chaperone
Source: J Biol Chem. 2025 Aug 13;301(9):110595. doi: 10.1016/j.jbc.2025.110595 (PMC12450639; doi:10.1016/j.jbc.2025.110595)
Supplement: Supporting information [file mmc1.docx]

**Supporting Information**

**Table S1.** Primers used for qPCR.

| **Transcript** | **Forward primer** | **Reverse primer** |
| --- | --- | --- |
| MAP2 | CCAGAAGTCCCCATGGCAAA | ACTCAGCTCCGCCATCATTC |
| TUBB3 | CCCAGCTTTGCTTCTCACCA | TTCTCTGCCTTGGACTCACG |
| NR1 | ATGCGCGTCTACAACTGGAA | TTCTCTGCCTTGGACTCACG |
| NR2A | ACGTCTACCTTCTTCCAGTTTGG | GCCAGTCATAATCCTGCATGATCT |
| NR2B | CCCAGTCTGGTGGCAGGG | GCAGCAGTGGTGATTATGGCA |

**Figure S1. HT22 differentiation.** *A***,** Transcript analysis for mature neuron-specific markers MAP2 and TUBB3. HT22 cells were either undifferentiated or differentiated for 24h in Neurobasal media containing 1X N2 supplement and 2mM L-glutamine. Human brain microvascular cells (EC) were used as a non-neuronal cell control. Data are expressed as fold-change in transcript compared to undifferentiated HT22. Statistical significance was calculated using one-way ANOVA and Dunnett’s multiple comparisons test compared to undifferentiated HT22; *, *p* < 0.05; **, *p* < 0.01; ***, *p* < 0.001; ****, *p* < 0.0001. *B*, Transcript analysis for glutamatergic NMDA receptor subunits. HT22 cells were either undifferentiated or differentiated for 24h as in panel A. Data are expressed as fold-change in transcript compared to undifferentiated HT22. Statistical significance was calculated using two-tailed t-test; ***, *p* < 0.001; ****, *p* < 0.0001. *C*, Immunocytochemistry staining (63X) of mature neuron markers in differentiated HT22 cells. Cells were labeled with antibodies for MAP2 (green), β-III-tubulin (green), or NeuN (red) and stained with Hoechst (blue). Cells were also stained with only secondary antibodies and Hoechst as a control for non-specific secondary antibody binding. Single channel images were adjusted for brightness and contrast using ImageJ. A 20X image was collected for cells incubated with IgG control antibody to determine non-specific antibody isotype staining.

**Figure S2. Cell viability in response to menadione treatment.** Differentiated HT22 cells were treated with menadione at the indicated concentrations for 24 h. After 24 h, media samples were collected and LDH release was measured. Data were normalized to the untreated control. Statistical significance was determined using one-way ANOVA with Dunnett’s multiple comparisons test to compare to the untreated control; **, p < 0.01; ***, p < 0.001.
